# Hong Kong high school students' perceptions of the new secondary school curriculum

**DOI:** 10.3389/fped.2022.881515

**Published:** 2022-07-22

**Authors:** Diya Dou, Daniel T. L. Shek

**Affiliations:** Department of Applied Social Sciences, The Hong Kong Polytechnic University, Kowloon, Hong Kong SAR, China

**Keywords:** NSS, Hong Kong, high school student, educational reform, academic performance, noncognitive skills

## Abstract

**Background:**

The New Senior Secondary (NSS) curriculum in Hong Kong aims to change the exam-oriented culture and promote students' all-around development. This reform emphasizes student-centered learning and promotes a shift from a top-down approach to school-based management, with the ultimate goal to help students become lifelong learners. This study examined students' perceptions of the NSS curriculum with a focus on their noncognitive development (e.g., self-understanding, positive values, purpose in life, and resilience).

**Methods:**

The data were collected from 3,498 Secondary 6 students in Hong Kong (Girls: 47.7%; Mean age: 17.33 years) using a self-reported questionnaire in 2015. We examined the psychometric properties of the instrument, “Perceptions of the New Secondary School Curriculum” (PNSC), and conducted multigroup CFA to evaluate the measurement invariance of PNSC across genders. Paired *t*-test analysis was used to examine whether students perceived the junior and senior secondary curricula differently. A series of multivariate analysis of variance (MANOVA) were conducted to examine students' perceptions of the curriculum by gender and by academic performance level.

**Results:**

Results based on percentage responses showed that most students liked the curriculum and acknowledged its benefits in promoting their noncognitive development. However, substantial proportions of the students also reported relatively negative responses to some items, particularly their fondness for senior secondary education. Students generally reported higher fondness for the junior secondary curriculum than for the senior secondary curriculum. Girls had more positive perceptions of the NSS curriculum than did boys. High-performing students liked the NSS curriculum the most and perceived the most benefits of the curriculum in promoting their noncognitive skills, whereas low-performing students showed the lowest levels of fondness for/interest in the curriculum and perceived benefits.

**Conclusions:**

Our findings support previous evidence showing initial success in promoting students' noncognitive skills but also alert educators and policymakers that the curriculum should not leave the low-performing students behind. Collective efforts from schools, educational bureaus, researchers, and policymakers are needed to take appropriate measures to cater to students' balanced development.

## Introduction

The education system in Hong Kong is characterized by its morbid emphasis on academic excellence indexed by public examination results. Students scoring highly in major public examinations are considered “successful.” Universities in Hong Kong often publicize the number of students with high results they admit each year. Research revealed that more than 70% of the students felt worried about getting poor mathematics marks despite spending almost 40 h per school week on academic-related learning (1). In fact, Hong Kong students have been ranked top in several international assessments, such as Programme for International Student Assessment (PISA) and Trends in International Mathematics and Science Study (TIMSS), on major subjects, including reading, mathematics, and sciences (1, 2). However, despite such high academic achievement, which demonstrates Hong Kong students' solid cognitive ability, many aspects of their noncognitive skills are lagging in international competitions. According to the PISA 2003 report, Hong Kong students' average performance in mathematics ranked first among all participating countries, but their self-concepts and confidence in this subject were among the weakest (1). PISA 2018 revealed that Hong Kong students' resilience and problem-solving skills were ranked below the OECD average and those of many Asian countries, including Korea, Vietnam, and Malaysia (2).

In response to the above criticism and the need to build twenty-first-century skills in adolescents, the New Senior Secondary (NSS) curriculum was implemented in Hong Kong in 2009, aiming to change the exam-oriented culture and promote students' all-around development through a more flexible learning system. This reform emphasizes student-centered learning and prioritizes the concept of “learning to learn,” which provides the fundamental strategy to promote students' “independent learning, whole-person development, and lifelong learning” (3, 4). This reform enables a shift from a top-down approach to school-based management and from external intervention to increasing resources for student-centered pedagogies at the school level (5).

Although the NSS reform aims to benefit students' overall development, including their cognitive skills (e.g., critical thinking and reasoning) and noncognitive skills (e.g., identity, positive values, social awareness, and resilience), this assumption still lacks empirical support (4). Notably, previous studies evaluating the effectiveness of the NSS curriculum have mainly focused on the achievement of learning outcomes (e.g., critical thinking, learning motivation, and reading habits) while paying relatively less attention to the development of students' noncognitive skills. Additionally, students' perceptions of the NSS curriculum reflect their satisfaction with the curriculum and should be considered an essential outcome indicator of reform assessment. However, whether students are satisfied with the NSS curriculum is a question that remains to be answered. Using survey data collected from 3,498 students in 28 secondary schools in Hong Kong, this study attempted to understand students' perceptions of the NSS curriculum, particularly their noncognitive development.

### The context of NSS reform in Hong Kong

The NSS curriculum was officially launched in 2009. As a bold step forward for secondary education in Hong Kong, the NSS reform adopted several significant changes to the high school education system. The NSS curriculum reduced the total number of secondary school years (from 7 to 6 years) and increased the number of tertiary education curriculum years (from 3 to 4 years). As for public examination, a single Hong Kong Diploma of Secondary Education Examination (HKDSE) at the end of Secondary 6 replaced the previous two examinations: the Hong Kong Certificate of Education Examination and the Hong Kong Advanced Level examination. Guided by the framework and related learning goals, significant changes under the NSS curriculum consisted of designating Liberal Studies as one core subject and introducing an elective subject system and Other Learning Experience (OLE) as key elements of the curriculum (6). The core subject, Liberal Studies, creates opportunities for students to study and reflect on contemporary social and cultural issues concerning personal and social development, which would broaden students' knowledge base and increase their citizenship (6). The elective subjects include practical courses such as Engineering and Production, Business, Accounting and Financial Studies to prepare students for future career development. Additionally, courses such as Ethics and Religious Studies, Moral and Civic Education, and Community Service are provided to improve students' lifelong skills and to inspire their full potential and whole-person development.

The flexible and practical structure of the NSS curriculum allows schools to organize activities to develop students' noncognitive skills (4). The core subject, Liberal Studies, covers topics under the themes “self and personal development,” “society and culture,” and “science, technology, and the environment” for students to understand themselves as well as their relationships with society and the environment (7). Liberal Studies is expected to improve students' self-understanding, social awareness, and responsibility through investigating a variety of social issues (8–10). The introduction of OLE explicitly reflects the learning goals of the NSS curriculum, which stress the development of students' generic skills, values, and attitudes. The OLE provides various vocational development education and related life planning training activities for students to gain understanding of their potential and find purpose in life (11). Under the OLE, each student needs to spend 405 hours of extra-curricular activities in five learning areas (Moral and Civic Education, Community Service, Career-related Experiences, Aesthetic Development, and Physical Development) and complete Student Learning Profile (SLP). In practice, some schools organized a variety of aesthetic and physical activities, such as dance classes, to strengthen students' confidence, perseverance, resilience, and positive coping strategies (4). Along with the subject offer, the NSS curriculum also encourages a shift in teaching philosophies and methods to promote students' noncognitive development. Teachers are expected to be the key enactors to ensure students have “opportunities for developing diverse ways of learning according to their interests, needs and abilities to achieve the aims of education” (3). For example, reflexivity is a central pedagogical principle that is particularly emphasized in inquiry-based learning activities (8). Teachers applying reflexivity are aware of the influence of personal perspectives in constructing “knowledge” and thus more likely to support students to reflect in-depth and develop personal opinions (12).

At the initial stage of the reform, researchers identified various challenges schools and teachers face in translating government policy into school-based practices and implementing innovative curricula in real classrooms, including teachers' heavy workloads, limited understanding of the reform, insufficient professional training, and students' learning diversity (13, 14). Research has also demonstrated that effective school leadership and management are vital in gauging successful curriculum reform. For example, Lai and Cheung (15) interviewed school principals and found that shared instructional leadership and decision-making contributed to effective school-based curriculum implementation.

Following the first round of completion (i.e., 2009–2015), researchers have a more comprehensive picture to examine the NSS curriculum implementation. Empirical studies have shown that effective and examination-oriented practices coexist in curriculum implementation (9, 10). Studies on effective practices further identified effective school practices, such as collaborative inquiry, instructional strategy, and catering to learner diversity (16). However, some gaps between the ambitious objectives and the actual implementation exist. For example, although teachers concur with the reform objectives to promote students' critical thinking, they face pedagogical challenges, such as limited updated resources, which could hinder their support for students (9, 10). Additionally, there is limited empirical evidence supporting the hypothesis that the NSS reform promotes students' noncognitive development. Existing research has mainly focused on skills closely related to academic learning, including positive attitudes of learning and self-concepts regarding academic work (4). Among the limited studies on this topic, Cheung et al. (17) examined some aspects of students' noncognitive development, such as communication skills and problem-solving. They revealed that students perceived themselves as performing well in establishing good interpersonal relationships but as weak in self-regulated and reflective learning. Chan et al. (18) examined the influence of the NSS curriculum on students' transition from school to the work market and found an improvement in students' generic competencies. Yuen et al.'s (4) study based on 8,122 secondary school students indicated that moderate progress had been made in students' overall performance in generic skills, positive values, and attitudes over the key learning areas. Thus, further examination of different aspects of noncognitive skills against the backdrop of the NSS reform is needed.

### Research gaps

The first research gap involves a lack of validated measurements of students' perceptions of the NSS curriculum in the existing literature. Primarily, most measures of academic or school satisfaction have been developed in the West. Moreover, imported measures have rarely been validated in the Chinese context (19). Obviously, a lack of validated assessment tools on perceptions of the NSS curriculum would hinder meaningful analyses of the effectiveness of the educational reform. Only Cheung et al. (17) developed and validated a scale to examine different stakeholders' perceptions of the NSS curriculum. As the items of Cheung et al.'s scale were related to the policy recommendations and reform objectives, this scale mainly focused on the quality of student learning under the NSS curriculum but did not cover attributes including self-understanding, resilience, emotional competence, and purpose in life, which are profoundly critical for nurturing students' positive development in the long run (19). Against the above background, this study attempted to examine the psychometric properties of the “Perceptions of the New Secondary School Curriculum” (PNSC). This scale was developed to measure four dimensions of Hong Kong students' perceptions of the NSS curriculum, including their fondness for and interest in the junior/senior secondary curriculum and perceived benefits of the junior/senior secondary curriculum in promoting positive and holistic development. Shek and Chai (20) conducted exploratory factor analysis (EFA) and confirmatory factor analysis (CFA) to examine the structure of the scale. In the present study, we further examined the convergent, discriminant, and factorial validities (i.e., factorial invariance) of the scale.

The second research gap involves a lack of investigation of students' voices over the educational reform in the literature. Most existing studies have mainly adopted the perspectives of school heads and teachers as they are the frontline reformers at the school level (i.e., expert views). Due to the top-down nature of educational reforms, research has often focused on identifying on-site difficulties; the discrepancies between the understandings of the public, school, and policymakers; and characteristics of effective schooling (4, 17, 21). Students' perceptions of the curriculum and school programs reflect their academic satisfaction, which is an essential indicator of educational transformation success (20, 22). Marchiondo et al. (23) defined academic satisfaction as “the attraction or positive feelings that a student associates with the college or program in question” (p. 610). Scholars also found a positive relationship between academic satisfaction and students' developmental outcomes. For example, Rowe, Stewart, and Patterson (24) found that positive learning experiences in schools could strengthen students' health and well-being. On the contrary, students who do not like school or feel disconnected from school tend to have lower academic performance or even drop out (25, 26). However, the voice of secondary students, who are the direct recipients of educational services and major stakeholders, has received insufficient attention. Gaps might also exist between students' and other stakeholders' perceptions (4, 20). Among the limited studies examining students' experiences, Cheung et al. (17) used a large and representative sample to examine students' perceptions. They asked students to indicate how well the students in their schools performed in some aspects of noncognitive development (e.g., growth mindset and communication skills). However, how students individually perceive their own development was not examined. Additionally, the survey did not provide sufficient information about how students perceive the benefits of the NSS curriculum, as it mainly focused on the “objective” outcomes instead of perceived evaluation.

The third research gap is that it is not clear whether students perceive the junior and senior curricula differently. Some studies revealed a decreasing trend in school satisfaction reported by students (27). One possible reason is that students' academic stress might gradually increase when they reach the senior secondary level. In particular, senior high school students in Hong Kong face heavy academic stress due to the shortened school year and the competitive final examination under the NSS curriculum. Research on Chinese students revealed higher levels of perceived academic stress reported by students in higher grades (e.g., senior secondary school years) than junior secondary students (28). Zhu and Shek (29) examined the perceived effectiveness of a school-based curriculum promoting positive youth development in the Chinese context. They found that students in junior grades showed significantly more positive evaluations than did students in senior grades. However, other studies have not found a close correlation between grade and school satisfaction (30). Nevertheless, there is a need to understand the potential difference between students' perceptions of the junior and senior secondary curricula.

The fourth research gap concerns a lack of investigation of gender differences in students' perceptions of the NSS curriculum. Previous studies on school and academic satisfaction revealed that girls tended to report higher engagement and satisfaction with school than did boys (31). However, gender differences were not observed in other studies. For example, Løhre et al.'s study conducted with 149 boys and 119 girls revealed no gender differences in school well-being (32). Similarly, some studies based on Chinese students did not find any gender differences in children's perceived school satisfaction (33). Because this issue is not well addressed under the NSS curriculum, further examination is needed.

The fifth research gap is that, despite the good intention for the NSS curriculum to change the exam-oriented system and promote all students' noncognitive skills, we have little knowledge of whether high- and low-performing students differ in their perceptions of the benefits of the NSS curriculum in promoting their noncognitive development. Theoretically speaking, the NSS curriculum should apply the “no loser” principle in Hong Kong's education and promote whole-person development of all students despite their academic performance levels. As the new system introduces more selective and practical subjects, low-achieving students should have more opportunities to develop their potential in different aspects and could benefit more from the system than high-performing students. However, empirical studies have shown inconclusive findings concerning the relationship between students' performance and their satisfaction with school. Previous studies revealed that high-performing students often received higher levels of teacher expectation and support, reported more positive school experiences, and benefitted more from the school curriculum, which might contribute to more positive perceptions of the school curriculum (34, 35). For example, Zhou et al.'s (36) study, conducted with Chinese high school students, revealed that high-achieving students perceived higher levels of need satisfaction at schools, which in turn improved their future academic achievement. Similar results were found in the context of Hong Kong that gifted students with high performance reported higher levels of satisfaction than average students did (37). On the other hand, high-performing students in Chinese contexts often exhibit perfectionism (37) and experience heavier social expectations (38), which could negatively influence their perceptions of the school curriculum. Nevertheless, empirical evidence examining the relationship between students' performance and their perceptions of the NSS curriculum is scarce, thus calling for further studies addressing this research gap.

This study attempted to answer the following research questions to address the above research gaps:

Research Question 1: What are the psychometric properties of the PNSC as a measure of the students' perceptions of the NSS curriculum?

Research Question 2: What are high school students' perceptions of the NSS curriculum indexed by the PNSC?

Research Question 3: Do students perceive the junior and senior secondary school curricula differently? As the school work and examination pressure are often heavier in senior secondary studies than in junior secondary studies (39), we hypothesized that perceptions of the junior secondary curriculum would be more positive than those of the senior secondary curriculum (Hypothesis 1).

Research Question 4: Do boys and girls differ in their perceptions of the secondary school experience? Based on previous findings (31, 40), we expected that girls would have more favorable perceptions of the secondary school curriculum than boys would (Hypothesis 2).

Research Question 5: Do the perceptions of the NSS curriculum differ among students with different academic and school performance? Based on previous findings of studies conducted in the Chinese contexts (36, 37), we hypothesized that students with better academic and school performance would have more favorable perceptions of the NSS curriculum. If the NSS curriculum really emphasizes both academic and nonacademic skills, students with different academic performance levels would likely not differ appreciably in either direction in their perceptions. It is also possible that low-achieving students would perceive more benefits from the NSS curriculum because it seeks to change the role of examination scores as the only criterion of student performance. Therefore, we explored the relationships between students' academic performance and their perceptions of the NSS curriculum without making hypotheses.

## Method

### Participants and procedure

This study is a part of a 6-year longitudinal project entitled Positive Adolescent Training through Holistic Social Programs (P.A.T.H.S.) initially launched in the 2009/2010 school year. The data used in the present study were collected every year between 2009/2010 and 2015/2016. This project aimed to examine adolescent development of high school students in Hong Kong, including their positive youth development, family functioning, academic performance, and perceptions of the NSS curriculum. Participants were from 28 schools in different areas of Hong Kong, including Kowloon, New Territories, and Hong Kong Island. All students in Grade 7 of the 28 schools were invited to participate in this study. A questionnaire was administered to participants annually for six consecutive years across secondary school education. Students responded to the questionnaire in classrooms during school hours. Written informed consent was obtained from students, parents, and school administrators before the data collection. Researchers provided information about the purpose, confidentiality, and anonymity principles of the research to all involved parties. The project was reviewed and approved by the authors' university.

In the present study, we used a sample of 3,498 Secondary 6 students who participated in the sixth wave of data collection in 2016 (i.e., the last year of the NSS curriculum). Among them, 1,669 were girls (47.7%), 1,821 were boys (52.1%), and 9 did not report their gender (0.2%). The mean age of the participants was 17.33 years.

### Instruments

#### Perspectives of the new secondary school curriculum (PNSC) scale

The research team developed the PNSC to measure students' perceptions and experiences of the new curriculum during secondary education based on a six-point scale (1 = “Strongly Disagree” and 6 = “Strongly Agree”), with higher levels of satisfaction indicated by higher scores. As reported in a previous study (20), the final scale contained 18 items and reflected a four-factor structure, which included (1) fondness for and interest in the junior secondary curriculum (4 items, e.g., “I like the new junior secondary curriculum”), (2) perceived benefits of the junior secondary curriculum in promoting positive and holistic development (5 items, e.g., “The junior curriculum can help me establish positive values and attitudes”), (3) fondness for and interest in the senior secondary curriculum (4 items), and (4) perceived benefits of the senior secondary curriculum in promoting positive and holistic development (5 items).

#### Academic and school performance

The Academic and School Competences Scale developed by Shek and Yu (41) was used to assess students' self-reported school performance. This scale includes three items: “How do you perceive your academic performance as compared with classmates?” “Are you satisfied with your current academic performance?” and “How do you perceive your school conduct?” A five-point Likert scale was used (1 = very poor/very unsatisfied, 5 = very good/very satisfied). The scale has been validated in the context of Hong Kong and was used in previous studies (42). In the present study, this scale showed accepted reliability (Cronbach's α = 0.639; mean inter-item correlatio*n* = 0.370).

#### Analysis

We used SPSS 26.0 and the lavaan package in R software (43) to examine the psychometric properties of the PNSC (Research Question 1). We first tested the normality of the PNSC by examining the skewness and kurtosis of all items under the scale. According to Curran et al. (44), the requirements of normality should be met if the maximum likelihood estimation is adopted for CFA. Following the Fornell-Larcker testing system (45), we also assessed the convergent validity and discriminant validity of the scale. The convergent validity was assessed by average variance extracted (AVE) and composite reliability (CR), which should exceed 0.50 and 0.70, respectively, to gauge good convergent validity (45). Additionally, discriminant validity was examined by comparing the square root of the AVE for each factor and inter-construct correlations involving the factor (45). If the square root of the AVE of factors is greater than all inter-construct correlations, the discriminant validity is supported. Internal consistency of the instrument was examined by calculating Cronbach's alpha, McDonald's omega, and mean inter-item correlations for the scale and factors. In addition to the previous factor analysis findings (20), we conducted multigroup CFA (MGCFA) to evaluate measurement invariance across gender. Following the steps suggested by van de Schoot et al. (46), we conducted measurement invariance tests on a series of CFA models, including configural, metric, scalar, and error variance invariance models. The goodness-of-fit indices adopted in the present study included comparative fit index (CFI), non-normed fit index (NNFI), root mean square error of approximation (RMSEA), and standardized root mean square residual [SRMR, (46, 47)]. As Kline (47) suggested, a satisfactory model fit requires CFI and NNFI values to be above 0.90, RMSEA to be lower than 0.08, and SRMR to be smaller than 0.08. Given the large sample size, we adopted difference-in-CFI (ΔCFI ≤ 0.01) as the main indicator for measurement invariance tests because the χ^2^ test is sensitive to sample size and model complexity (46).

Concerning the response profiles (Research Question 2), results of the descriptive statistics analysis (e.g., frequency responses) were reported. Paired *t*-test analysis was used to examine whether students perceived the junior and senior secondary curricula differently (Research Question 3). A series of MANOVAs were conducted to examine students' perceptions of the curriculum by gender (Research Question 4). Bonferroni correction was adopted in interpreting the results. As four factors of the PNSC were included as dependent variables in the analyses, we adjusted the significance level to 0.013 (0.05/4). Finally, to examine whether students' academic performance is related to their perceptions of the curriculum (Research Question 5), we conducted a MANOVA with Bonferroni correction to compare the perceptions among students with high (+1 SD), moderate (mean), and low (−1 SD) levels of perceived academic performance.

## Results

### Psychometric properties of the PNSC

We first examined the skewness and kurtosis of all items in the PNSC to test the normality. According to Curran et al. (44), the absolute values of skewness and kurtosis should be lower than 2 and 7, respectively, so that the requirement of normality can be met and maximum likelihood estimation would be appropriate for CFA. Results showed that for items in the PNSC subscales, absolute values of skewness ranged between 0.277 and 0.691, which were all below 2. In addition, absolute values of kurtosis ranged from 0.009 to 0.613, which were lower than 7. Therefore, all items can be considered to demonstrate a normal distribution (44).

The means, SD, and correlations of variables are summarized in Table 1. We found significant correlations between the four factors of the PNSC (*r*s ranged between 0.552 and 0.772, *p* < 0.01, see Table 1). We used AVE and CR to assess the convergent validity (45). As shown in Table 2, the AVE values for all four factors were greater than 0.5, and the CR values were above 0.70, supporting the good convergent validity of the scale (45). Following Fornell and Larcker's recommendations (45), we assessed the discriminant validity of the scale by comparing the square root of the AVE for each factor and the correlation involving the factor. The results showed that the values of the square root of the AVE for each factor ranged between 0.889 and 0.914, which were larger than the correlations under investigation ranging from 0.552 to 0.772. Thus, the discriminant validity of the scale was supported. Results of the reliability test showed good internal consistency of the PNSC (Cronbach's alpha = 0.967, McDonald's omega = 0.967, see Table 2) and all four factors (Cronbach's alpha values ranged between 0.946 and 0.953, McDonald's omega values ranged between 0.947 and 0.952, see Table 2).

**Table 1:** Results of correlation analysis among research variables.

		**Mean**	**SD**	**1**	**2**	**3**	**4**	**5**	**6**
1	Age	17.20	0.58						
2	Gender ^a^			0.017					
3	Academic and school performance	2.95	0.65	−0.026	0.003				
4	Fondness for and interest in the junior secondary curriculum (4 items)	3.71	1.07	0.059**	0.044*	0.159**			
5	Perceived benefits of the junior secondary curriculum (5 items)	3.71	1.01	0.102**	0.049*	0.198**	0.772**		
6	Fondness for and interest in the senior secondary curriculum (4 items)	3.51	1.15	0.052*	−0.020	0.274**	0.568**	0.572**	
7	Perceived benefits of the senior secondary curriculum (5 items)	3.72	1.09	0.065**	0.033	0.236**	0.552**	0.704**	0.772**

^a^Male = 1, Female = 2. *p <0.05, **p <0.01.

**Table 2:** Reliability and validity of full scale and factors of PNSC.

		**No. of item**	**Cronbach's alpha**	**McDonald's omega**	**Mean inter-item correlation**	**AVE**	**CR**
Factor 1	Fondness for and interest in the new junior secondary curriculum	4	0.949	0.950	0.826	0.835	0.953
Factor 2	Perceived benefits of the new junior secondary curriculum	5	0.952	0.951	0.800	0.791	0.950
Factor 3	Fondness for and interest in the senior secondary curriculum	4	0.946	0.947	0.815	0.824	0.949
Factor 4	Perceived benefits of the senior secondary curriculum	5	0.953	0.952	0.802	0.793	0.950
Full scale	PNSC	18	0.967	0.967	0.625		

*AVE, Average Variance Extracted; CR, Composite Reliability*.

### CFA and measurement invariance tests

Based on the work of Shek and Chai (20), we performed CFA on the 4-factor PNSC model with the entire sample. Results revealed that the baseline model (Model 0) demonstrated a good fit {χ^2^_(121)_ = 1,852.567; CFI = 0.977; NNFI = 0.971; RMSEA = 0.065 [90% CI: 0.063 to 0.068]; SRMR = 0.024}. The satisfactory fit provided basis for performing multigroup CFA to examine measurement invariance across genders.

Multigroup CFA were conducted on a subsample of boys (*N* = 1,821) and a subsample of girls (*N* = 1,669). The baseline model (Model 0) was tested separately by gender in both subsamples to gauge its factorial stability (48). The baseline model demonstrated a good fit to the data in both the boy subsample {χ^2^_(121)_ = 1,038.85; CFI = 0.978; NNFI = 0.972; RMSEA = 0.066 [90% CI: 0.062–0.07]; SRMR = 0.022} and the girl subsample {χ^2^_(121)_ = 1,162.212; CFI = 0.968; NNFI = 0.96; RMSEA = 0.073 [90% CI: 0.069–0.077]; SRMR = 0.031}, illustrating the factorial stability in both subsamples.

Next, we tested the configural invariance model (Model 1), metric invariance model (Model 2), scalar invariance model (Model 3), and error variance invariance model (Model 4). As shown in Table 3, Model 1 showed adequate fit to the data {χ^2^_(242)_ = 2,201.062; CFI = 0.974; NNFI = 0.967; RMSEA = 0.07 [90% CI: 0.067–0.072]; SRMR = 0.025}, supporting the invariance of the factorial structure across the boy and girl subsamples. In Model 2, factor loadings were constrained to be the same across genders {χ^2^_(256)_ = 2,223.99; CFI = 0.973; NNFI = 0.968; RMSEA = 0.068 [90% CI: 0.065–0.07]; SRMR = 0.025}. As shown in Table 3, results of the χ^2^ tests revealed significant differences (Δχ^2^ = 22.93, Δ*df* = 14, *p* < 0.01) between Models 1 and 2, between Models 2 and 3 (Δχ^2^ = 55.83, Δ*df* = 14, *p* < 0.001), and between Models 3 and 4 (Δχ^2^ = 150.07, Δ*df* = 18, *p* < 0.001). As suggested by Cheung and Rensvold (49), we referred to the value of difference-in-CFI (ΔCFI ≤ 0.01) instead of changes in χ^2^ due to the large sample size. The comparison between Model 2 and Model 1 yielded a ΔCFI (<0.001) below 0.01, suggesting invariance in factor loadings across genders. In Model 3, both factor loadings and measurement intercepts were assumed to be equal across genders {χ^2^_(270)_ = 2,279.824; CFI = 0.973; NNFI = 0.969; RMSEA = 0.067 [90% CI: 0.064–0.069]; SRMR = 0.026}. The value of ΔCFI was below 0.01, suggesting invariance in measurement intercepts across the two subsamples (see Table 3). Lastly, Model 4 constrained factor loading, measurement intercept, and the error variance of each variable to be equal across the boy and girl subsamples {χ^2^_(288)_ = 2,429.895; CFI = 0.971; NNFI = 0.969; RMSEA = 0.067 [90% CI: 0.064–0.069]; SRMR = 0.026}. The value of ΔCFI (0.002, see Table 3) was below 0.01, denoting the invariance of measurement error for each item across genders.

**Table 3:** Summary of goodness-of-fit for CFA and invariance tests (Multigroup comparisons by gender).

**Model no**.	**Model description**	**Comparative model**	χ^2^	Δχ^2^	***df***	Δ***df***	***p*** **-value**	**CFI**	Δ**CFI**	**NNFI**	Δ**CFI** ≤ **|0.01|?**	**RMSEA (90% CI)**
0	Baseline model	–	1,852.57	–	121	–	–	0.977	–	0.971		0.065
												(0.063–0.068)
1	Configural invariance	–	2,201.06	–	242	–	–	0.974	–	0.967	–	0.070 (0.067–0.072)
2	Metric invariance	–	2,223.99	–	256	–	–	0.973	–	0.968	–	0.068 (0.065–0.07)
		2 vs. 1	–	22.93	–	14	*p* < 0.01	–	0.001	–	Yes	–
3	Scalar invariance	–	2,279.82	–	270	–	–	0.973	–	0.969	–	0.067 (0.064–0.069)
		3 vs. 2	–	55.83	–	14	*p* < 0.001	–	0.000	–	Yes	–
4	Error variance invariance	–	2,429.90	–	288	–	–	0.971	–	0.969	–	0.067 (0.064–0.069)
		4 vs. 3	–	150.07	–	18	*p* < 0.001	–	0.002	–	Yes	–

*N_whole_= 3,490; N_males_= 1,821; N_females_= 1,669; CFI: Comparative Fit Index; RMSEA: Root Mean Square Error of Approximation; CI: confidence interval; Δχ^2^: change in χ^2^ compared to the previous model; Δdf: change in degrees of freedom compared to the previous model; ΔCFI: change in CFI compared to the previous model; ΔCFI ≤ |0.01|?; Model 0 = Baseline model using the whole sample; Model 1 = no equality constraints were imposed; Model 2: equality constraints were imposed on all factor loadings; Model 3: equality constraints were imposed on all factor loadings and intercepts of the measured variables; Model 4: equality constraints were imposed on all factor loadings, intercepts, and residual variance*.

### Profiles of perceptions based on the PNSC

The percentage responses of students to the items on the PNSC are summarized in Tables 4, 5 for the junior and senior secondary curricula, respectively. Concerning the junior secondary curriculum, the majority of students liked the curriculum (67.38%) and agreed that the curriculum was interesting (66.04%), encouraged them to reflect (59.15%), and enhanced learning interests (63.46%). Students also acknowledged the benefits of the curriculum in promoting their positive and holistic development. For example, more than two-thirds of the participants agreed that the junior curriculum helped them establish their values and deepen their self-understanding.

**Table 4:** Percentages of responses to the questions on academic satisfaction with the junior curriculum.

**No**.	**Item**	**Valid N (%)**	**Mean**	**SD**	**Strongly disagree (%)**	**Disagree (%)**	**Slightly disagree (%)**	**Slightly agree (%)**	**Agree (%)**	**Strongly agree (%)**	**Negative responses**^a^ **(%)**	**Positive responses**^b^ **(%)**
Factor 1: Fondness for and interest in the new junior secondary curriculum												
1	The junior curriculum is	3,470	3.70	1.16	214	327	619	1,523	686	101	1,160	2,310
	interesting.	(99.2%)			(6.12%)	(9.35%)	(17.7%)	(43.54%)	(19.61%)	(2.89%)	(33.16%)	(66.04%)
2	The junior curriculum	3,472	3.58	1.15	230	355	818	1,397	589	83	1,403	2,069
	encourages me to reflect.	(99.26%)			(6.58%)	(10.15%)	(23.38%)	(39.94%)	(16.84%)	(2.37%)	(40.11%)	(59.15%)
3	The junior curriculum	3,470	3.67	1.17	228	321	701	1,431	700	89	1,250	2,220
	enhances my learning interest.	(99.2%)			(6.52%)	(9.18%)	(20.04%)	(40.91%)	(20.01%)	(2.54%)	(35.73%)	(63.46%)
4	I like the junior	3,470	3.78	1.20	220	292	601	1,437	766	154	1,113	2,357
	curriculum.	(99.2%)			(6.29%)	(8.35%)	(17.18%)	(41.08%)	(21.9%)	(4.4%)	(31.82%)	(67.38%)
Factor 2: Perceived benefits of the new junior secondary curriculum												
5	The junior curriculum	3,466	3.78	1.09	188	216	655	1,617	684	106	1,059	2,407
	helps me establish my values.	(99.09%)			(5.37%)	(6.17%)	(18.72%)	(46.23%)	(19.55%)	(3.03%)	(30.27%)	(68.81%)
6	The junior curriculum	3,471	3.79	1.10	185	238	622	1,617	699	110	1,045	2,426
	deepens my self-understanding.	(99.23%)			(5.29%)	(6.8%)	(17.78%)	(46.23%)	(19.98%)	(3.14%)	(29.87%)	(69.35%)
7	The junior curriculum	3,473	3.68	1.12	201	272	791	1,468	645	96	1,264	2,209
	improves my ability to deal with adverse situations.	(99.29%)			(5.75%)	(7.78%)	(22.61%)	(41.97%)	(18.44%)	(2.74%)	(36.13%)	(63.15%)
8	The junior curriculum	3,470	3.64	1.15	218	293	830	1,404	617	108	1,341	2,129
	improves my emotional competence.	(99.2%)			(6.23%)	(8.38%)	(23.73%)	(40.14%)	(17.64%)	(3.09%)	(38.34%)	(60.86%)
9	The junior curriculum	3,460	3.56	1.22	288	343	807	1,307	605	110	1,438	2,022
	helps me find my purpose in life.	(98.91%)			(8.23%)	(9.81%)	(23.07%)	(37.36%)	(17.3%)	(3.14%)	(41.11%)	(57.8%)

^a^*Negative responses, Strongly disagree + disagree + slightly disagree; ^b^Positive responses, Slightly agree + agree + strongly agree*.

**Table 5:** Percentages of responses to the questions on academic satisfaction with the senior secondary curriculum.

		**Valid N (%)**	**Mean**	**SD**	**Strongly disagree (%)**	**Disagree (%)**	**Slightly disagree (%)**	**Slightly agree (%)**	**Agree (%)**	**Strongly agree (%)**	**Negative responses**^a^ **(%)**	**Positive responses**^b^ **(%)**
Factor 3: Fondness for and interest in the new senior secondary curriculum												
1	The curriculum is	3,491	3.48	1.26	321	429	812	1,193	641	95	1,562	1,929
	interesting.	(99.8%)			(9.18%)	(12.26%)	(23.21%)	(34.11%)	(18.32%)	(2.72%)	(44.65%)	(55.15%)
2	The curriculum	3,491	3.60	1.24	292	352	733	1,298	700	116	1,377	2,114
	encourages me to reflect.	(99.8%)			(8.35%)	(10.06%)	(20.95%)	(37.11%)	(20.01%)	(3.32%)	(39.37%)	(60.43%)
3	The curriculum	3,491	3.50	1.27	330	402	816	1,169	673	101	1,548	1,943
	enhances my learning interest.	(99.8%)			(9.43%)	(11.49%)	(23.33%)	(33.42%)	(19.24%)	(2.89%)	(44.25%)	(55.55%)
4	I like the senior	3,489	3.39	1.29	393	434	863	1,117	579	103	1,690	1,799
	secondary curriculum.	(99.74%)			(11.23%)	(12.41%)	(24.67%)	(31.93%)	(16.55%)	(2.94%)	(48.31%)	(51.43%)
Factor 4: Perceived benefits of the new senior secondary curriculum												
5	The senior secondary	3,483	3.67	1.18	252	278	743	1,394	719	97	1,273	2,210
	curriculum helps me establish my values.	(99.57%)			(7.2%)	(7.95%)	(21.24%)	(39.85%)	(20.55%)	(2.77%)	(36.39%)	(63.18%)
6	The senior curriculum	3,487	3.75	1.19	234	271	655	1,412	794	121	1,160	2,327
	deepens my self-understanding.	(99.69%)			(6.69%)	(7.75%)	(18.72%)	(40.37%)	(22.7%)	(3.46%)	(33.16%)	(66.52%)
7	The senior curriculum	3,487	3.70	1.21	253	300	709	1,356	733	136	1,262	2,225
	improves my ability to deal with adverse situations.	(99.69%)			(7.23%)	(8.58%)	(20.27%)	(38.77%)	(20.95%)	(3.89%)	(36.08%)	(63.61%)
8	The senior curriculum	3,484	3.61	1.24	282	340	769	1,278	684	131	1,391	2,093
	improves my emotional competence.	(99.6%)			(8.06%)	(9.72%)	(21.98%)	(36.54%)	(19.55%)	(3.74%)	(39.77%)	(59.83%)
9	The senior curriculum	3,469	3.72	1.30	310	296	627	1,248	806	182	1,233	2,236
	helps me find my purpose in life.	(99.17%)			(8.86%)	(8.46%)	(17.92%)	(35.68%)	(23.04%)	(5.2%)	(35.25%)	(63.92%)

^a^*Negative responses, Strongly disagree + disagree + slightly disagree; ^b^Positive responses, Slightly agree + agree + strongly agree*.

As for the senior secondary curriculum, students demonstrated neutral to positive attitudes toward the curriculum. Slightly more than half of the students liked the curriculum (51.43%) and agreed that the curriculum was interesting (55.15%) and enhanced learning interests (55.55%). Around 60% of students believed the curriculum encouraged self-reflection. As for the perceived benefits of the senior secondary curriculum, students perceived that the curriculum helped them establish values (63.18%), deepen self-understanding (66.52%), improve the ability to deal with adverse situations (63.61%), improve emotional competence (59.83%), and find their purpose in life (63.92%).

On the other hand, although a significant proportion of the respondents acknowledged the benefits of the curriculum in promoting their noncognitive development in both junior and senior secondary education, substantial proportions reported relatively negative perceptions. Concerning the junior secondary curriculum, around one third of students stated negative perceptions (see Table 4). For example, 40.11% of students disagreed that the curriculum encouraged them to reflect. More than 41% of students generally disagreed that the curriculum helped them find their life's purpose. As for the senior secondary curriculum, about 40% of students gave negative responses on items under fondness for the curriculum, and one third generally disagreed on items under perceived benefits of the curriculum. For example, more than 48% of students gave negative responses to the item “I like the senior secondary curriculum.” Around 40% of students generally disagreed that the senior curriculum improved their emotional competence.

### Differences in perceptions of junior and senior secondary curricula

As displayed in Table 6, results of paired *t*-tests showed that students had more positive perceptions of the junior secondary curriculum (mean = 3.682) than of the senior secondary curriculum (mean = 3.493; *t* = 10.779, *p* < 0.001, Cohen's *d* = 0.183). However, no significant difference was observed in perceived benefits between junior and senior secondary education (*t* = 0.235, *p* = 0.814). Generally, students liked the junior secondary curriculum more than the senior secondary curriculum, supporting Hypothesis 1.

**Table 6:** Results of paired *t*-test between students' perceptions of the junior and senior secondary curriculum.

	**Junior**		**Senior**		**95% CI**		***t***	***df***	***p***	**Cohen's** ***d***
	**Mean**	**SD**	**Mean**	**SD**	**Lower**	**Upper**				
Fondness for and interest in the curriculum	3.682	1.088	3.493	1.174	0.155	0.224	10.779***	3,475	0.000	0.183
Perceived benefit of the curriculum	3.691	1.041	3.688	1.123	−0.024	0.031	0.235	3,474	0.814	0.004

***p <0.001.

### Gender differences in the PNSC

We conducted a MANOVA to examine gender differences in subscales of the PNSC. We first tested the homogeneity of covariance matrices with Box's M test. The result of Box's M test was 145.266 with a *p*-value <0.001, which was interpreted as significant according to the cutoff suggested by Huberty and Petoskey (50). Thus, this assumption was violated. As researchers have argued, Box's M test is sensitive to large sample sizes and could detect very small departures from homogeneity (51). Following the recommendation of Tabachnick et al. (51), we adopted Pillai's trace criterion as it is the most robust statistic for protection against departures from the assumptions. MANOVA results showed that girls generally possessed more positive perceptions of the curriculum than did boys [Pillai's trace = 0.011, *F*_(4, 3643)_ = 10.068, *p* < 0.001, η^2^ = 0.011, see Table 7], which generally supported Hypothesis 2. Girls showed higher levels of fondness for and interest in the junior secondary curriculum (*F* = 13.58, *p* < 0.001, η^2^ = 0.004). However, we found no gender difference in fondness for and interest in the senior secondary curriculum. With regard to the perceived benefits of the curriculum, a significant gender difference was observed for both junior and senior secondary curricula. As compared with boys, girls perceived more benefits of the junior secondary curriculum (*F* = 15.37, *p* < 0.001, η^2^ = 0.004) and the senior secondary curriculum (*F* = 10.52, *p* < 0.01, η^2^ = 0.003).

**Table 7:** Results of MANOVA by gender.

	**Boy** **Mean (SD)**	**Girl** **Mean (SD)**	***F***	*η^2^**p***
Academic satisfaction of the new curriculum	3.60 (1.05)	3.70 (0.85)	10.07***	0.011
Factor 1: Fondness for and interest in junior curriculum	3.62 (1.19)	3.75 (0.96)	13.58***	0.004
Factor 2: Perceived benefits of junior curriculum	3.62 (1.14)	3.76 (0.92)	15.37***	0.004
Factor 3: Fondness for and interest in senior curriculum	3.49 (1.26)	3.50 (1.08)	0.00	0.000
Factor 4: Perceived benefits of senior curriculum	3.63 (1.21)	3.76 (1.02)	10.52**	0.003

**p <0.01, ***p <0.001.

### The role of student academic and school performance

We performed a MANOVA to test potential mean differences between academic performance and PNSC scores. Similarly, Box's M test was performed to check the homogeneity of covariance matrices. Based on Huberty and Petoskey's guideline (50), we reported Pillai's trace in the MANOVA as a significant value of Box's M test was observed. We found a significant multivariate effect for the four factors of perceptions of the NSS curriculum based on students' perceived academic performance [Pillai's trace = 0.058, *F*_(8, 4, 010)_ = 14.973, *p* < 0.001, η^2^ = 0.029]. As shown in Table 8, MANOVA results showed that students' academic performance levels were positively related to all subscales of perceptions of the NSS curriculum. Additionally, results of *post-hoc* comparisons revealed significant differences between all pairs in comparisons (*p* < 0.001). That is, as compared with the other two groups, high-performing students liked the NSS curriculum the most, whereas low-performing showed the lowest levels of fondness for/interest in the curriculum. We found the same patterns when comparing the perceived benefits of the NSS curriculum by academic performance. Notably, the mean score of low-performing students' fondness for/interest in the senior school curriculum was 2.82 (SD = 1.18), suggesting that they slightly disliked the senior secondary curriculum.

**Table 8:** Results of MANOVA by academic performance.

	**Low-performing** **Mean (SD)** **(*****N** =* **204)**	**Average-performing** **Mean (SD)** **(*****N** =* **1416)**	**High-performing** **Mean (SD)** **(*****N** =* **390)**	***F***	*η^2^**p***
Academic satisfaction of the new curriculum				15.19^***^	0.029
Factor 1: Fondness for and interest in junior curriculum	3.40 (1.24)	3.70 (1.03)	3.91 (1.04)	15.51^***^	0.015
Factor 2: Perceived benefits of junior curriculum	3.30 (1.12)	3.71 (0.98)	3.92 (1.00)	25.41^***^	0.025
Factor 3: Fondness for and interest in senior curriculum	2.82 (1.18)	3.52 (1.11)	3.87 (1.11)	58.12^***^	0.055
Factor 4: Perceived benefits of senior curriculum	3.18 (1.19)	3.72 (1.04)	4.01 (1.09)	39.98^***^	0.038

***p <0.001.

## Discussion

The NSS curriculum has been implemented for more than 10 years since its first launch in 2009. It aims to change the traditional examination-oriented educational ideology and enhance whole-person development and lifelong skills in high school students (52). In the past years, the Education Bureau (EDB) in Hong Kong kept reviewing the effectiveness of the NSS curriculum at the macro level by focusing on issues including teacher workload, students' diverse learning needs, curriculum design and implementation. The Task Force on Review of School Curriculum was set up in 2017 to holistically review the NSS curriculum. There are ongoing discussions among the public, researchers, schools, and policymakers about whether the missions have been achieved. This study contributes to the discussion by examining students' perceptions of the NSS curriculum, focusing on noncognitive attributes, using a validated scale and a large sample.

In addition to the previous factor analysis findings based on EFA and CFA (20), we further validated the scale evaluating students' perceptions of the NSS curriculum in this study. The findings underscored the good psychometric properties of the scale. First, the PNSC and the subscales showed good internal consistency. Second, the results suggested good convergent validity and discriminant validity of the PNSC. Third, the findings supported the four-factor structure identified in the previous study (20) with good factorial invariance across gender. The large sample of the present study also improved the generalizability of the results. Valid measurements are fundamental for scientific research, particularly for research examining latent variables. This scale can be used as a valid instrument in future studies examining students' perceptions of the NSS curriculum.

Results of the response profiles showed that most students generally liked the junior secondary curriculum (positive response rates of items >59%) and perceived the curriculum as beneficial to promoting their positive attributes, particularly in establishing values and self-understanding (positive response rates of items >57%). Similar results were found for students' perceptions of the senior secondary curriculum. Nevertheless, despite the existence of the positive response profiles, two observations should be highlighted. First, it is notable that nearly half of the students (48.31%) gave negative responses to the item “I liked the senior secondary curriculum.” This might be related to the heavier workload and pressure in senior secondary education than in junior secondary education (53). Senior secondary students often report heavy pressure as they have much to learn, need to do well on exams and make choices about their career, and often feel worried about the future (39). The NSS curriculum doubtlessly increases students' perceived uncertainty despite schools' and teachers' efforts to prepare them psychologically. Second, students gave relatively more positive responses to most items under perceived benefits of junior secondary education than those of senior secondary education (e.g., positive values, self-understanding, and emotional competence), except for the item “The curriculum helps me find my purpose in life.” The positive response rates for this item were 57.8 and 63.9% for junior and senior secondary education, respectively. This might be attributed to the introduction of applied learning courses and OLE in senior secondary education, which offer students opportunities to gain knowledge, values, and career-related skills in different areas and further promote students' career aspirations and orientation for lifelong development in their areas of interest (54). As revealed in existing literature, purpose in life is associated with positive developmental outcomes and negatively related to problem behaviors (55). Research shows that purposeful adolescents are generally happier and report stronger academic self-concepts (56).

The comparisons between the junior and senior secondary curricula further revealed a significant difference between students' fondness for the junior and senior secondary curricula. Students presented a neutral attitude regarding their fondness for/interest in the senior secondary curriculum (mean = 3.49) and a positive attitude toward the junior secondary curriculum (mean = 3.62). Our results echo previous findings revealing a decreasing trend in school satisfaction reported by students (27), particularly in the Chinese contexts (28). One explanation is that the new subjects and assessments are officially applied to the senior secondary curriculum but not to the junior secondary curriculum. Therefore, students might feel that the senior secondary curriculum is more challenging than before. The HKDSE could also lead to pressure in their studies. Interestingly, we found no significant difference between students' perceived benefits of the junior and senior secondary curricula. As discussed earlier, students in senior grades often experience heavy pressure due to the shortened school year and the competitive final examination. The findings are inconsistent with previous studies reporting higher perceived benefits for junior grades than those for senior grades (29). We could argue that the senior secondary curriculum reform might be successful in promoting positive development for students, which is in line with previous findings (17).

As for gender differences, results showed that girls generally possessed more positive perceptions of the secondary curriculum than did boys, which is in line with previous findings (31). A possible explanation is that girls tend to perceive more support from schools and teachers, which could contribute to their subjective feelings toward the school curriculum and life (57). Wang, Meissel (58) conducted a study with 1,199 Chinese high school students and found that teachers tended to have higher expectations of girls than of boys, which often led to more positive school experiences and achievement in girls. Empirical studies in Hong Kong also suggest that girls are generally more engaged and satisfied with school than boys are (40). It is noteworthy that we observed no gender difference in the fondness for and interest in the senior secondary curriculum. A possible explanation is that although girls tend to be more engaged than boys, they often experience higher levels of academic stress or are more likely to be negatively influenced by stressors (59). Thus, the increased academic stress in senior secondary education could shape girls' perceptions of the school and even negatively affect their fondness for and interest in the curriculum.

There are two observations regarding the perceptions of the NSS curriculum among students with different performance levels. The first observation concerns the positive relationship between students' academic performance and their perceptions of the NSS curriculum. That is, high-performing students liked the curriculum the most and perceived more benefits from it than did the moderate- and low-performing groups; low-performing students liked the NSS curriculum the least and perceived fewer benefits of the curriculum in promoting their positive attributes. One possibility is that some courses adopted advanced pedagogies and materials, such as inquiry-based learning, which require relatively high levels of students' cognitive and metacognitive competence to ensure successful implementation (8). Thus, high-performing students with stronger cognitive competence might adapt better to the new teaching and learning patterns, in particular at the early stage of the implementation when teachers are facing significant pedagogical challenges and heavy workload in this paradigm shift. As revealed in previous studies, teachers perceived student diversity as a major obstacle and were not confident in applying the inquiry-oriented approach (9, 14). In addition, although high-achieving students also perceived heavy academic pressure under the NSS curriculum, previous studies revealed that high-achieving students often possess stronger academic resilience, self-efficacy, and confidence when facing academic stress (60). The second observation is that low-performing students generally disliked the senior secondary curriculum (mean = 2.83). When facing academic challenges, low-performing students are more likely to develop negative self-beliefs in studies and to be unable to cope with future examinations, which could contribute to the low levels of fondness for and interest in the senior secondary curriculum. Evidence showed that students' academic stress is not eased but rather intensified under the NSS curriculum (53). The adaptation of the single examination (HKDSE) for the entire secondary school stage, the shortened years for senior secondary study, and the introduction of new curricula that are much deeper and more comprehensive in terms of knowledge and skills acquisition could add to low-performing students' learning frustration and dislike of the senior secondary curriculum.

## Implications and conclusions

Students' perceptions of the school curriculum are an important indicator of education reform success. Students' negative experience and evaluation of the curriculum would decrease their learning motivation and engagement, which would adversely affect their academic achievement. Moreover, as school life plays a significant role in student development, satisfaction with the school curriculum (i.e., academic well-being) is an important aspect of adolescent well-being (20). Lower levels of academic well-being are often associated with other negative learning outcomes, such as academic anxiety and low levels of self-efficacy and performance (61). In addition, students who perceive limited benefits of the curriculum for their noncognitive development tend to face challenges in developing important positive attributes such as resilience and purpose in life, which could lead to a sense of hopelessness and even depression when suffering from heavy academic stress (62). In their studies on life skills education in Hong Kong high schools, Shek et al. (63) found that students recognized the importance of life skills (e.g., emotional competence and resilience) but perceived insufficient education in the formal school curriculum. Their observations suggest a need for promoting life skills education in the NSS curriculum, which is also in line with the policy recommendation made by the Task Force on Review of School Curriculum (64) that schools should further promote students' social developmental needs.

The present study suggests that gaps exist between objectives, design, and the actual implementation of the NSS curriculum, which is common or even inevitable for educational reforms (14). The results showed that students generally acknowledged the benefits of the NSS curriculum in their noncognitive development. However, about one third of students indicated less satisfactory perceptions. In addition, low-performing students reported slight feelings of dislike toward the senior secondary curriculum. There is room for refining the curriculum and supporting low-performing students to achieve overall development.

Some scholars claimed that the NSS curriculum enlarged students' academic achievement gap (53). The present study provides some insights into this observation by revealing low-performing students' low levels of fondness for the senior secondary curriculum. As revealed in the literature, students' subjective perceptions of the school curriculum are closely related to their intrinsic learning motivation, self-efficacy, and school satisfaction, which in turn promote their academic performance as well as positive attributes (20). If low-performing students dislike the curriculum and do not perceive many benefits of the curriculum for their noncognitive development, they might feel disappointed with the curriculum reform and even be left behind in the long run. Previous studies have revealed that students with lower levels of positive attributes such as resilience tend to perceive higher levels of academic stress and lower life and school satisfaction (60). Moreover, empirical evidence showed that teachers tended to underestimate the ability of low-achieving students across the school year (65). Teachers might have less confidence in low-achieving students adapting to the NSS curriculum, which could lead to these students having less favorable perceptions of the curriculum. Collective efforts from schools, educational bureaus, researchers, and policymakers are needed to support teacher training, curriculum design, and assessment to assure the successful reform implementation for all students. A quasi-experimental study by Jong et al. (66) reported that their well-designed game application adopted in Liberal Studies had positive effects on high-, moderate-, and low-performing students. Notably, they found the largest effect sizes of the positive influence for low-performing students. On the contrary, teachers without sufficient support tended to stick to previous teaching patterns or assessment measurements due to the heavy workload and limited resources (9). Additionally, although different activities are taking place at schools, the implementation requires alignment between the curriculum design and actual provision. Studies revealed that some key OLE elements were not found in the current NSS curriculum, such as the curriculum to enhance career planning and life purpose (11). A recent survey conducted by the Education Bureau (EDB) in 2021 revealed that although the recommend time allocation for the core subjects were 45–55%, many schools allocated more lesson time to these subjects and students lacked diversified learning experiences (67). As suggested in the final report by the Task Force on Review of School Curriculum (64), more flexibility should be provided for schools to plan activities and adapt teaching and learning to the new paradigm. Additionally, schools should take appropriate measures to cater to students' balanced development; in particular, they should pay more attention to their social developmental needs. In short, although our findings support previous evidence suggesting preliminary success of the NSS curriculum in promoting students' noncognitive skills, the present findings also alert educators and policymakers that the curriculum should not leave the low-performing students behind.

Several limitations of the study should be noted. First, we only used the data collected from Secondary 6 students at Wave 6 to gain relatively comprehensive perceptions of the curriculum. It would be informative to use longitudinal data in future studies, particularly when examining the relationships between students' perceptions of the curriculum, their actual positive youth development, and academic performance. As revealed in previous research, these factors might influence each other reciprocally (68). Second, we used perceived academic and school performance instead of grades or teachers' evaluations because the related information is confidential (42). Future studies might consider using grades if the information is available. Third, we did not explore the potential differences between the perceived benefits of diverse noncognitive skills. As revealed in the literature, students reported the progress for self-concepts as less favorable than those for technology competence and reading habits (4). It is possible that the perceived benefits would differ among different noncognitive skills. Further examinations on this issue would help frontline teachers and schools to adjust the materials and pedagogies to promote balanced development in different aspects of noncognitive skills. Fourth, the data were collected between 2009/2010 and 2015/2016, and students' perceptions might have changed in recent years. Nevertheless, it is comforting to know that the present findings echo the observations highlighted in the report by the Task Force on Review of School Curriculum published in 2020, which served as the directional recommendations for the refinement of the NSS curriculum in the near future. Nevertheless, collecting longitudinal data of students' perceptions of the curriculum in future is important for curriculum evaluation and refinement.

## Data availability statement

The raw data supporting the conclusions of this article will be made available by the authors, without undue reservation.

## Ethics statement

The studies involving human participants were reviewed and approved by Human Subjects Ethics Sub-committee of The Hong Kong Polytechnic University. Written informed consent to participate in this study was provided by the participants' legal guardian/next of kin.

## Author contributions

Conceptualization, methodology, writing—review and editing, project administration, and funding acquisition: DD and DS. Formal analysis and writing—original draft preparation: DD. Supervision: DS. Both authors have read and agreed to the published version of the manuscript.

## Funding

The Project P.A.T.H.S. was financially supported by the Hong Kong Jockey Club Charities Trust. This paper was financially supported by Wofoo Foundation and the start-up grant of the DD (Project ID: P0035101).

## Conflict of interest

The authors declare that the research was conducted in the absence of any commercial or financial relationships that could be construed as a potential conflict of interest.

## Publisher's note

All claims expressed in this article are solely those of the authors and do not necessarily represent those of their affiliated organizations, or those of the publisher, the editors and the reviewers. Any product that may be evaluated in this article, or claim that may be made by its manufacturer, is not guaranteed or endorsed by the publisher.

## References

[1] OECD. Learning for Tomorrow's World: First Results from Pisa 2003. Berlin, Germany: OECD Publishing (2004).

[2] Schleicher A. Pisa 2018: Insights and Interpretations. Berlin, Germany: OECD Publishing (2019).

[3] Curriculum Development Council. Learning to Learn: The Way Forward in Curriculum Development. Hong Kong: Government Printer (2000).

[4] Yuen TWW Cheung ACK Wong PM. A study of the impact of the first phase of the curriculum reform on student learning in Hong Kong. Int J Educ Manage. (2012) 26:710–28. 10.1108/09513541211263782

[5] Cheng YC Mok MMC. School-based management and paradigm shift in education: an empirical study. Int J Educ Manage. (2007) 21:517–42. 10.1108/09513540710780046

[6] Fung C-L Yip W-Y. The policies of reintroducing liberal studies into Hong Kong Secondary Schools. Educ Res Policy Pract. (2009) 9:17–40. 10.1007/S10671-009-9076-3

[7] Deng Z. The formation of a school subject and the nature of curriculum content: an analysis of liberal studies in Hong Kong. J Curriculum Stud. (2009) 41:585–604. 10.1080/00220270902767311

[8] Adrian YKC. Challenges of cultivating critical citizens in Hong Kong voices from liberal studies pre-service teachers. Asian Educ Dev Stud. (2019) 8:14–27. 10.1108/AEDS-06-2017-0050

[9] Fung D Lui W-M Liang T Su A. The way forward for the development of liberal studies: how teachers perceive its introduction and implementation in Hong Kong Secondary Schools. Asia Pac Educ Rev. (2017) 18:123–34. 10.1007/S12564-017-9471-Z

[10] Fung D Lui WM. Is liberal studies a political instrument in the secondary school curriculum? Lessons from the umbrella movement in Post-Colonial Hong Kong. Curriculum J. (2017) 28:158–75. 10.1080/09585176.2016.1275727

[11] Lee JCK. Curriculum reform and supporting structures at schools: challenges for life skills planning for secondary school students in China (With Particular Reference to Hong Kong). Educ Res Policy Pract. (2017) 16:61–75. 10.1007/S10671-016-9202-Y

[12] Brownlee J Schraw G Berthelsen D. Personal Epistemology and Teacher Education. London, UK: Routledge (2012). 10.4324/9780203806616

[13] Cheung ACK Wong PM. Effects of school heads' and teachers' agreement with the curriculum reform on curriculum development progress and student learning in Hong Kong. Int J Educ Manage. (2011) 25:453–73. 10.1108/09513541111146369

[14] Fok PK. Liberal studies reform in Hong Kong secondary education: contrasting desirability with feasibility. Educ Res Policy Pract. (2016) 15:209–30. 10.1007/S10671-015-9190-3

[15] Lai E Cheung D. Implementing a new senior secondary curriculum in Hong Kong: instructional leadership practices and qualities of school principals. School Leader Manage. (2013) 33:322–53. 10.1080/13632434.2013.813459

[16] Keung CPC Cheung ACK Mak BSY Tam WWY. Perceptions of Hong Kong Secondary School teachers on effective pedagogical practices for curriculum reform: a multi-group analysis. Asia Pac J Educ. (2021) 15:1–13. 10.1080/02188791.2021.1988901

[17] Cheung ACK Keung CPC Mak BSY. Examining the key stakeholders' perceptions of student learning: towards a paradigm shift in secondary education in Hong Kong. Asia Pac J Educ. (2019) 39:532–47. 10.1080/02188791.2019.1604318

[18] Chan KO Ng MK So JC Chan VC. Evaluation of generic competencies among secondary school leavers from the new academic structure for senior secondary education in Hong Kong. Public Adm Policy. (2021). 10.1108/PAP-07-2020-0033

[19] Shek DTL Siu AM. Evaluation of a positive youth development program in Hong Kong: issues, principles and design. Int J Adolesc Med Health. (2006) 18:329–40. 10.1515/IJAMH.2006.18.3.329 17068914

[20] Shek DTL Chai W. The impact of positive youth development attributes and life satisfaction on academic well-being: a longitudinal mediation study. Front Psychol. (2020) 11:2126. 32982869 10.3389/fpsyg.2020.02126 PMC7490328

[21] Cheng YC Mok MMC. What effective classroom? Towards a Paradigm Shift. School Effect School Improvement. (2008) 19:365–85. 10.1080/0924345080253517434065817

[22] Wach FS Karbach J Ruffing S Brunken R Spinath FM. University students' satisfaction with their academic studies: personality and motivation matter. Front Psychol. (2016) 7:55. 10.3389/fpsyg.2016.00055 26909049 PMC4754397

[23] Marchiondo K Marchiondo LA Lasiter S. Faculty incivility: effects on program satisfaction of Bsn students. J Nurs Educ. (2010) 49:608–14. 10.3928/01484834-20100524-05 20509588

[24] Rowe F Stewart D Patterson C. Promoting school connectedness through whole school approaches. Health Educ. (2007). 10.1108/09654280710827920

[25] Archambault I Janosz M Morizot J Pagani L. Adolescent behavioral, affective, and cognitive engagement in school: relationship to dropout. J School Health. (2009) 79:408–15. 10.1111/j.1746-1561.2009.00428.x 19691715

[26] Shochet IM Dadds MR Ham D Montague R. School connectedness is an underemphasized parameter in adolescent mental health: results of a community prediction study. J Clin Child Adolesc Psychol. (2006) 35:170–9. 10.1207/S15374424jccp3502_1 16597213

[27] Malley J Basic J Beck M Tavra VK Feric M Conway J. Student perceptions of their schools: an international perspective. Int J Real Ther. (2003) 23:4–11.

[28] Sun J Dunne MP Hou X-Y Xu A-Q. Educational stress among chinese adolescents: individual, family, school and peer influences. Educ Rev. (2013) 65:284–302. 10.1080/00131911.2012.659657

[29] Zhu X Shek DTL. Subjective outcome evaluation of a positive youth development program in Mainland China. Res Soc Work Pract. (2021) 31:285–97. 10.1177/104973152098080235600112

[30] Huebner ES Ash C Laughlin JE. Life experiences, locus of control, and school satisfaction in adolescence. Soc Indic Res. (2001) 55:167–83. 10.1023/A:1010939912548

[31] Tessema M Ready K Yu WH. Factors affecting college students' satisfaction with major curriculum: evidence from nine years of data. Int J Human Soc Sci. (2012) 2:10.

[32] Løhre A Moksnes UK Lillefjell M. Gender differences in predictors of school wellbeing? Health Educ J. (2014) 73:90–100. 10.1177/0017896912470822

[33] Hui EK Sun RC. Chinese Children's perceived school satisfaction: the role of contextual and intrapersonal factors. Educ Psychol. (2010) 30:155–72. 10.1080/01443410903494452

[34] Baker JA. Teacher-student interaction in urban at-risk classrooms: differential behavior, relationship quality, and student satisfaction with school. Elem Sch J. (1999) 100:57–70. 10.1086/461943

[35] Verkuyten M Thijs J. School satisfaction of elementary school children: the role of performance, peer relations, ethnicity and gender. Soc Indic Res. (2002) 59:203–28. 10.1023/A:1016279602893

[36] Zhou J Huebner ES Tian L. The reciprocal relations among basic psychological need satisfaction at school, positivity and academic achievement in chinese early adolescents. Learn Instruct. (2021) 71:101370. 10.1016/j.Learninstruc.2020.101370

[37] Fang L Yuen M Fung E Zhang J Chan S Wu F. Life satisfaction of gifted and average adolescents in Hong Kong: validation of the Chinese Brief Multidimensional Students' Life Satisfaction Scale (Bmslss). Appl Res Qual Life (2022) 17:751–61. 10.1007/S11482-021-09932-8

[38] Chen X Cheung HY Fan X Wu J. Factors related to resilience of academically gifted students in the Chinese cultural and educational environment. Psychol Sch. (2018) 55:107–19. 10.1002/Pits.22044

[39] Kouzma NM Kennedy GA. Self-reported sources of stress in senior high school students. Psychol Rep. (2004) 94:314–6. 15077784 10.2466/pr0.94.1.314-316

[40] Yuen CY. Linking life satisfaction with school engagement of secondary students from diverse cultural backgrounds in Hong Kong. Int J Educ Res. (2016) 77:74–82. 10.1016/j.Ijer.2016.03.003

[41] Shek DTL Yu. L. Internet addiction in Hong Kong Adolescents: Profiles and Psychosocial Correlates. (2012) 2012:9. 10.1100/2012/104304 18690381 PMC5848730

[42] Shek DTL Li X. Perceived school performance, life satisfaction, and hopelessness: a 4-year longitudinal study of adolescents in Hong Kong. Soc Indic Res. (2016) 126:921–34. 10.1007/S11205-015-0904-Y 26912946 PMC4751166

[43] Rosseel Y. Lavaan: An R package for structural equation modeling and more. Version 05–12 (Beta). J Stat Softw. (2012) 48:1–36. 10.18637/jss.V048.i02

[44] Curran PJ West SG Finch JF. The robustness of test statistics to nonnormality and specification error in confirmatory factor analysis. Psychol Methods. (1996) 1:16. 10.1037/1082-989X.1.1.16

[45] Fornell C Larcker DF. Evaluating structural equation models with unobservable variables and measurement error. J Market Res. (1981) 18:39–50. 10.1177/002224378101800104

[46] Van de Schoot R Lugtig P Hox J A. Checklist for testing measurement invariance. Eur J Dev Psychol. (2012) 9:486–92. 10.1080/17405629.2012.686740

[47] Kline RB. Principles and Practice of Structural Equation Modeling. 4th ed. New York: The Guilford Press (2015).

[48] Byrne BM. Structural Equation Modeling With Mplus: Basic Concepts, Applications, and Programming. London, UK: Routledge (2013).

[49] Cheung GW Rensvold RB. Evaluating goodness-of-fit indexes for testing measurement invariance. Struct Equ Model. (2002) 9:233–55. 10.1207/S15328007SEM0902_522551991

[50] Huberty CJ Petoskey MD. Multivariate Analysis of Variance and Covariance. In: Handbook of Applied Multivariate Statistics and Mathematical Modeling. Amsterdam: Elsevier (2000). p. 183–208.

[51] Tabachnick BG Fidell LS Ullman JB. Using Multivariate Statistics: Pearson Boston, MA (2007).

[52] Wong PWL. Textbook Evaluation: A Framework for Evaluating the Fitness of the Hong Kong New Secondary School (Nss) Curriculum. Hong Kong, China: City University of Hong Kong (2011).

[53] Yu Y-C Yin H-B. Dilemmas of inclusive practice under the new senior secondary curriculum reform in Hong Kong. Educ J. (2016) 44:183–217.

[54] Curriculum Development Council. Progress Report on the New Academic Structure Review: The New Senior Secondary Learning Journey—Moving Forward to Excel (2013).

[55] Shek DTL. Life meaning and purpose in life among chinese adolescents: what can we learn from Chinese studies in Hong Kong? In: Wong PTP, Editor. The Human Quest for Meaning: Theories, Research, and Applications. London, UK: Routledge/Taylor & Francis Group (2012). p. 335–55.

[56] Hill PL Burrow AL Sumner R. Addressing important questions in the field of adolescent purpose. Child Dev Perspect. (2013) 7:232–6. 10.1111/Cdep.1204812521399

[57] Sax LJ Bryant AN Harper CE. The differential effects of student-faculty interaction on college outcomes for women and men. J Coll Stud Dev. (2005) 46:642–57. 10.1353/csd.2005.0067

[58] Wang S Meissel K Rubie-Davies CM. Teacher expectation effects in chinese junior high schools: exploring links between teacher expectations and student achievement using a hierarchical linear modelling approach. Soc Psychol Educ. (2021) 24:1305–33. 10.1007/S11218-021-09654-7

[59] Liu Y Lu Z. Chinese high school students' academic stress and depressive symptoms: gender and school climate as moderators. Stress Health. (2012) 28:340–6. 10.1002/smi.2418 22190389

[60] Borman GD Overman LT. Academic resilience in mathematics among poor and minority students. Element School J. (2004) 104:177–95. 10.1086/499748

[61] Lei W Zhang H Deng W Wang H Shao F Hu W. Academic self-efficacy and test anxiety in high school students: a conditional process model of academic buoyancy and peer support. Sch Psychol Int. (2021) 42:616–37. 10.1177/01430343211039265

[62] Shek DTL Liang L-Y. Psychosocial factors influencing individual well-being in Chinese adolescents in Hong Kong: a six-year longitudinal study. Appl Res Qual Life. (2018) 13:561–84. 10.1007/S11482-017-9545-4 30174758 PMC6105256

[63] Shek DTL Lin L Ma CMS Yu L Leung JTY Wu FKY. Perceptions of adolescents, teachers and parents of life skills education and life skills in high school students in Hong Kong. Appl Res Qual Life. (2020) 16:1847–60. 10.1007/S11482-020-09848-9

[64] Task Force on Review of School Curriculum. Optimise the Curriculum for the Future, Foster Whole-Person Development and Diverse Talents. Hong Kong: Government Printer (2020).

[65] Wang S Rubie-Davies CM Meissel K. The stability and trajectories of teacher expectations: student achievement level as a moderator. Learn Individ Differ. (2020) 78:101819. 10.1016/j.Lindif.2019.101819

[66] Jong MS-Y Chan T Hue M-T Tam VW. Gamifying and mobilising social enquiry-based learning in authentic outdoor environments. J Educ Technol Soc. (2018) 21:277–92.

[67] Education Bureau,. Optimising the Four Senior Secondary Core Subjects (2021). Available online at: https://www.edb.gov.hk/en/curriculum-development/renewal/opt_core_subj.html (accessed on May 13, 2022).

[68] Ning HK Downing K. The reciprocal relationship between motivation and self-regulation: a longitudinal study on academic performance. Learn Individ Differ. (2010) 20:682–6. 10.1016/j.Lindif.2010.09.010

