# Supplementary material for: Hong Kong high school students' perceptions of the new secondary school curriculum
Source: Front Pediatr. 2022 Jul 22;10:881515. doi: 10.3389/fped.2022.881515 (PMC9354656; doi:10.3389/fped.2022.881515)
Supplement: Supplementary file 1 [file Table_1.docx]

Appendix 1. A summary of research gaps and questions.

| Research gaps | Research questions |
| --- | --- |
| 1. A lack of validated measurements of students’ perceptions of the NSS curriculum | 1. What are the psychometric properties of the PNSC as a measure of the students’ perceptions of the NSS curriculum? |
| 1. A lack of investigation of students’ voices over the educational reform | 1. What are high school students’ perceptions of the NSS curriculum indexed by the PNSC? |
| 1. Little knowledge of whether students perceive the junior and senior curricula differently | 1. Do students perceive the junior and senior secondary school curricula differently? |
| 1. A lack of investigation of gender differences in students’ perceptions of the NSS curriculum | 1. Do boys and girls differ in their perceptions of the secondary school experience? |
| 1. Little knowledge of whether high- and low-performing students differ in their perceptions of the | 1. Do the perceptions of the NSS curriculum differ among students with different academic and school performance? |
